# The effects of varying protein and energy intakes on the growth and body composition of very low birth weight infants

**DOI:** 10.1186/1475-2891-10-140

**Published:** 2011-12-29

**Authors:** Juan Antonio Costa-Orvay, Josep Figueras-Aloy, Gerardo Romera, Ricardo Closa-Monasterolo, Xavier Carbonell-Estrany

**Affiliations:** 1 Neonatal Unit, Hospital Clínic, IDIBAPS, Universitat de Barcelona, Barcelona, Spain; 2 Neonatal Unit, Montepríncipe Hospital, Madrid, Spain; 3 Neonatal Unit, Hospital Joan XXIII, Tarragona, Universitat Rovira i Virgili, IISPV, Tarragona, Spain

**Keywords:** Bioelectrical impedance analysis, Nutrition, Newborn

## Abstract

**Objective:**

To determine the effects of high dietary protein and energy intake on the growth and body composition of very low birth weight (VLBW) infants.

**Study design:**

Thirty-eight VLBW infants whose weights were appropriate for their gestational ages were assessed for when they could tolerate oral intake for all their nutritional needs. Thirty-two infants were included in a longitudinal, randomized clinical trial over an approximate 28-day period. One control diet (standard preterm formula, group A, n = 8, 3.7 g/kg/d of protein and 129 kcal/kg/d) and two high-energy and high-protein diets (group B, n = 12, 4.2 g/kg/d and 150 kcal/kg/d; group C, n = 12, 4.7 g/kg/d and 150 kcal/kg/d) were compared. Differences among groups in anthropometry and body composition (measured with bioelectrical impedance analysis) were determined. An enriched breast milk group (n = 6) served as a descriptive reference group.

**Results:**

Groups B and C displayed greater weight gains and higher increases in fat-free mass than group A.

**Conclusion:**

An intake of 150 kcal/kg/d of energy and 4.2 g/kg/d of protein increases fat-free mass accretion in VLBW infants.

## Introduction

The Nutrition Committee of the American Academy of Pediatrics suggests that, with optimal care and nutritional support, the growth rates of very low birth weight (VLBW) infants should be similar to those of fetuses of the same gestational age [1]. Nevertheless, despite advances in perinatal medicine and nutritional protocols [2,3], it has not been possible to achieve this rate of growth in neonatal care units [4-7]. Postnatal growth restriction is associated with an increased risk of poor neurodevelopmental outcomes [8-11], and inappropriate postnatal nutrition is an important contributor to growth failure [12,13]. The goal of obtaining appropriate intrauterine growth rates after birth has been successfully achieved with enriched diets, but these diets may lead to disproportionate increases in fat mass [14]. Energy supplied as carbohydrates is more effective than energy supplied as fats in sparing protein oxidation in enterally fed low birth weight (LBW) infants [15]. At isocaloric intakes, carbohydrates are more effective than fats in enhancing growth and protein accretion in enterally fed LBW infants [16]. However, a diet with high-energy and high-carbohydrate content also results in increased fat deposition [16].

To better define the macronutrient requirements of these infants, the increase in lean body mass should be taken into consideration in addition to weight gain. The ratio of lean body mass to fat mass in the weight gained depends on the protein and energy ratio in the diet. If energy and protein intakes are inappropriate, weight gain and the rate of increase in length and head circumference are reduced [17]; however, if protein intake is appropriate, a relatively higher energy intake may enhance the rate of increase in skinfold thickness [17], which suggests that the excess energy is stored as fat [18]. However, Fairey et al. [19] did not show any difference in the proportion of fat to lean tissue gained in groups with a higher protein-to-energy ratio (3.2 g/100 kcal vs. 2.6 g/100 kcal).

The aim of this study was to explore the effects of high protein (4.2 to 4.7 g/kg/day) and high energy (150 kcal/kg/day) intakes on the growth and body composition of VLBW infants. Our hypothesis was that supplemented formula would be well tolerated and would increase weight and lean body mass in preterm infants compared to newborns who did not receive supplementation.

## Subjects and Methods

### Subjects

Thirty-eight preterm (gestation of 32 weeks or fewer) newborns with weights below 1500 g and who were appropriate for gestational age were included in the study. The newborns were admitted to the neonatology ward of Hospital Clinic in Barcelona, Spain. Their baseline characteristics and complications of prematurity for each study group are shown in Table 1, while the compositions of the enteral diets of each study group are shown in Table 2. All of the newborns were free of any complications when they were enrolled in the study; further, they had recovered their birth weight and were gaining weight. At the beginning of the study, they received only enteral nutrition without IV perfusion. Mechanical ventilation and parenteral nutrition were discontinued at least five days before the beginning of the study. Exclusion criteria were intrauterine growth restriction, chromosomal abnormalities, malformations, chronic diseases or need for oxygen treatment. Written informed parental consent was obtained prior to enrollment in the study. The Neonatology Ethics Committee of the Hospital Clinic approved the study.

**Table 1:** Baseline characteristics and complications prior to the beginning of the study.

	Breastfed (n = 6)	Group A (n = 8)	Group B (n = 12)	Group C (n = 12)
**Characteristics of the newborns**				

Born in the hospital	5	8	11	9

Male gender	4	2	5	9

Cesarean section	4	7	11	8

1-min Apgar ≤4	1	3	2	2

5-min Apgar ≤8	3	4	2	2

Resuscitation (endotracheal intubation)	0	4	1	3

Prenatal corticosteroids	4	8	10	9

**Complications of the newborns**				

Respiratory distress syndrome	4	5	3	8

Mechanical ventilation	3	5	2	7

Patent ductus arteriosus	1	4	1	3

Sepsis	2	1	3	3

Necrotizing enterocolitis	2	0	0	0

Intraventricular hemorrhage	2	1	1	0

Parenteral nutrition > 7 days	2	3	3	3

**Table 2:** Composition of enteral diets

	Diet	Protein (g/kg/d)	Protein/energy ratio (g/100 kcal)	Fats (g/kg/d)	Carbohy-drates (g/kg/d)	Energy (kcal/kg/d)
**Breastfed** **(Group BM)**	Breast milk 160 ml/kg/d + Enfamil^®^ 4.5 g/kg/d	3.4	2.5	8.1	11.7	133: 10.2% protein 54.8% fat 35.2% carbohydrate

**Group A**	Alprem^®^ 160 ml/kg/d	3.7	2.8	6.6	13.6	129: 11.5% protein 46.2% fat 42.3% carbohydrate

**Group B**	Alprem^®^ 160 ml/kg/d + Promod^®^ 0.66 g/kg/d + Duocal^®^ 3.7 g/kg/d	4.2	2.8	7.5	16.3	149.5: 11.2% protein 45.2% fat 43.6% carbohydrate

**Group C**	Alprem^®^ 160 ml/kg/d + Promod^®^ 1.3 g/kg/d + Duocal^®^ 3.3 g/kg/d	4.7	3.1	7.45	16.1	149.9: 12.5% protein 44.7% fat 42.8% carbohydrate

- Alprem (Nestlé): in 100 g = 506 kcal, protein 14.5 g, carbohydrate 53.6 g, fat 26.0 g.

- Enfamil Human Milk fortifier (Nutricia): 1 vial = 5 mL = 5 g = 7.5 kcal, protein 0.55 g, carbohydrate < 0.3 g, fat 0.55 g.

- ProMod protein powder (Abbott): in 10 g = 42.4 kcal, protein 7.6 g, carbohydrate 1.0 g, fat 0.9 g.

- Duocal MCT (Nutricia): in 10 g = 12.4 kcal, carbohydrate 1.8 g, fat 0.58 g.

### Methods

Breastfed infants served as a reference group (group BM; n = 6). The macronutrient content of breast milk in this study (Table 2) was obtained using the reported data on milk from mothers of premature infants during early lactation [20]. Following the standard practice, the milk from the mother was enriched using Enfamil^® ^Human Milk Fortifier (Mead Johnson). Patients who were not breastfed were randomized to receive one of the three formulas detailed in Table 2. Randomization was performed by nurses who prepared the formula in the morning, using sealed envelopes, in blocks of 6. The nurses were the only individuals who knew the contents of the envelopes; however these nurses did not provide care for the infants. During the duration of the randomized trial, the blinding remained in intact, except for when sample sizes were calculated at the beginning of the study. Standard preterm formula, Alprem^® ^(Nestle), was given to the control group (group A; n = 8). The two experimental groups received high-energy and high-protein formulas with different energy-to-protein ratios: 150 kcal/kg/d with 4.2 g/kg/d of protein for group B (n = 12) and 150 kcal/kg/d with 4.7 g/kg/d of protein for group C (n = 12). ProMod^® ^(Abbott) and Duocal^® ^(SHS) were used to increase the protein and energy contents of the preterm formula.

The weight, length and head circumference of each infant were measured by the same investigator every week for four weeks (approximately 28 days). Electronic scales, accurate to 1 g, were used to weigh the subjects. Length and head circumference were measured using non-stretch tape. Z-scores were calculated for each infant, taking into account sex and post-menstrual age, by applying neonatal growth curves from Catalonia, Spain, which contain data obtained from more than 200,000 newborns [21]. Body mass index (BMI) was calculated using the following formula: weight (in kg)/length^2 ^(in m).

Body composition was measured via total body electrical impedance analysis (BIA) and was performed by a single investigator. Impedance and resistance were measured using a Bioscan Spectrum^® ^(Biológica Tecnología Médica, Ltd., Barcelona, Spain). The clinical methodology followed the recommendations of Tang et al. [22]. Skin electrodes were applied using the tetrapolar surface method. An 800-μA and 50-kHz alternating current was applied through these electrodes. The subjects were placed in a prone position with slight pelvic elevation, legs bearing weight through the anterior knees, with hips flexed at 30°. The knees were flexed at 30°, and the ankles were dorsiflexed at 70°. The arms were placed comfortably forward with forearms parallel to the long axis of the body. The arms were adducted at 45°, the elbows were flexed at 45° and the hands were comfortably extended. For distal limb positions, the voltage electrodes were placed so the lower edge of the electrode overlapped the proximal skin crease on the dorsal aspects of the wrist and ankle at the level of the styloid process and the medial malleolus, respectively. The current electrodes were positioned distal to the voltage electrodes at a center-to-center distance of 2 cm for the hand and 3 cm for the foot.

Total body water was determined using the equation described by Tang et al. [22]: Total body water = (0.016 + 0.674 × weight - 0.038 × weight^2 ^+ 3.84 foot length^2^)/resistance. A fat-free mass (FFM) value was then obtained using the following equation: FFM = total body water/water percentage of the FFM. The water percentage of the FFM was based on the studies of Fomon et al. [23] and Ziegler et al. [24]. Once the FFM was known, the fat mass (FM) was estimated as follows: FM = body weight - FFM.

### Study design

A longitudinal, interventional, randomized clinical trial was used. At the beginning of the study, the weights, lengths and head circumferences of the subjects with the corresponding Z-scores were determined, along with their BMIs, FMs and FFMs. Data regarding anthropometric values, gestational age at birth and previous illnesses were obtained from hospital records. For the four-week study period, the 32 patients fed artificial formula were randomly assigned to either high-energy and high-protein diets (groups B and C) or a standard energy and protein diet (group A). Serological control measures (serum glucose, protein, ammonia, pH, base excess, urea, cholesterol and triglyceride levels) were assessed once in each patient, during the third week of the study, to detect nutrition-related adverse effects in the three groups. The weights, lengths and head circumferences with their corresponding Z-scores, along with the BMIs, FMs and FFMs were determined on approximately Day 28 of the study and were considered the final values for the study.

### Statistics

The sample size of each group was calculated according to the hypothesis that supplemented formula would increase FFM accretion. When the first five cases without supplementation (group A) and the first five supplemented cases (groups B or C) were analyzed on the 21st day of the study, their FFM accretions were 15.09+/-2.14 and 19.85+/-4.15 g/kg/day, respectively. Using these preliminary data, the sample size to compare two independently observed means by a bilateral analysis with 80% power and an α-risk of 0.05 was calculated to be 12 cases per group (10 cases plus 2 for possible drop-outs). Therefore, a sample size of 12 cases was considered suitable for each of the three groups.

All the variables displayed normal distributions. The results were expressed as the means ± SD. Cross-sectional differences in anthropometric and body composition measurements among all groups (A, B and C) were tested by analysis of variance (ONEWAY and Scheffé's test for multiple comparisons of at-birth and at-beginning variables, and UNIANOVA with covariates for comparisons of results at the end of the intervention). In the UNIANOVA, the factor was the group, and the covariates were the initial corresponding figure and the duration in days of the intervention. If the p-value of the factor group was < 0.1, the UNIANOVA with covariates tests were repeated to see if a significant difference existed between any of the three paired comparisons (group A versus group B, group A versus group C and group B versus group C). A chi-squared test was used to analyze the significance of the differences between qualitative variables. Statistical analyses were performed using SPSS 13.0 (SPSS, Inc., Chicago, IL). The results were considered statistically significant at p < 0.05.

## Results

There were no significant differences between groups in baseline characteristics or in incidences of the following complications related to prematurity: patent ductus arteriosus, intracranial hemorrhage, hyaline membrane disease, sepsis and necrotizing enterocolitis (Table 1).

Energy intake up to 150 kcal/kg/d and protein intake up to 4.7 g/kg/d were well tolerated by all subjects from both the clinical and analytical points of view. Analytical data by groups are shown in Table 3. The only difference observed when comparing groups B and C with group A was that infants in groups B and C exhibited higher urea levels (p = 0.032).

**Table 3:** Analytical data by group.

		**Mean **± **SD**	p
**Urea (mg/dl)**	A	9.0 ± 1.9	0.032
	B	12.0 ± 6.6	
	C	17.2 ± 8.5	

**Protein (g/l)**	A	44.0 ± 2.0	0.755
	B	45.6 ± 5.6	
	C	46.9 ± 6.7	

**Ammonia (mcg/dl)**	A	114.4 ± 44.1	0.445
	B	112.3 ± 30.0	
	C	128.8 ± 28.5	

**Triglycerides (mg/dl)**	A	106.5 ± 58.9	0.930
	B	76.8 ± 16.3	
	C	72.7 ± 25.6	

**Cholesterol (mg/dl)**	A	102.9 ± 20.6	0.422
	B	115.1 ± 21.1	
	C	107.0 ± 21.4	

**pH**	A	7.38 ± 0.07	0.289
	B	7.39 ± 0.02	
	C	7.36 ± 0.05	

**Base excess (mmol/l)**	A	-0.14 ± 3.2	0.911
	B	0.23 ± 2.9	
	C	-0.38 ± 4.1	

Number of patients in each group: 8 in A, 12 in B and 12 in C.

One measurement was performed on one occasion for each patient in the 3rd week of the study.

**Figure 1:**
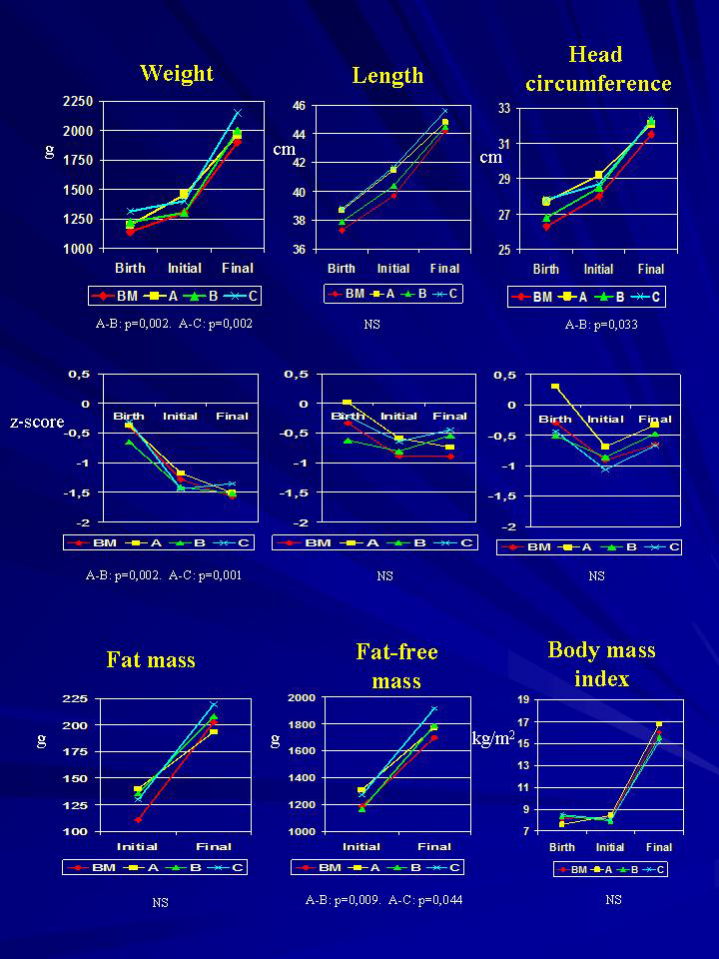
**Anthropometric measurement means at birth and at the beginning and the end of the study**.

The corrected ages of the preterm infants and measurements of the weights, lengths and head circumferences, along with the corresponding Z-scores, are shown in Table 4 and Figure 1. In addition, Table 4 and Figure 1 also show the FMs, FFMs and BMIs at birth and at the beginning and end of the study. Throughout the study, groups B and C exhibited increases in weight gain, Z-score of the weight gain, and FFM accretion. These changes were statistically significant for the factor group and for the covariates initial corresponding figure and duration of the intervention.

**Table 4:** Characteristics of the infants at birth and at the beginning and the end of the study

		At birth	p *	At the beginning	p *	At the end	p **
**Age (corrected** **gestational age**, **weeks)**	BM	29.0 ± 1.7	----	32.2 ± 2.3	-----	35.7 ± 1.94	----
	
	A	29.6 ± 1.6	0.683	32.8 ± 0.8	0.761	36.2 ± 0.60	0.406
	B	30.2 ± 1.4		32.6 ± 1.22		36.0 ± 1.02	
	C	29.8 ± 1.7		32.9 ± 1.70		36.4 ± 1.53	

**Weight (g)**	BM	1138 ± 173	----	1302 ± 173	----	1903 ± 223	----
	
	A	1196 ± 243	0.589	1452 ± 216	0.259	1967 ± 189	0.000
	B	1220 ± 221		1303 ± 213		1998 ± 146	A-B: 0.002
	C	1313 ± 336		1404 ± 189		2154 ± 202	A-C: 0.002
							B-C: 0.622

**Weight (Z-score)**	BM	-0.380 ± 0.918	----	-1.279 ± 0.984	----	-1.567 ± 0.738	----
	
	A	-0.368 ± 0.678	0.445	-1.170 ± 0.407	0.706	-1.501 ± 0.525	0.001
	B	-0.646 ± 0.476		-1.408 ± 0.639		-1.513 ± 0.605	A-B: 0.002
	C	-0.309 ± 0.819		-1.438 ± 0.970		-1.347 ± 1.091	A-C: 0.001
							B-C: 0.213

**Length (cm)**	BM	37.3 ± 2.2	----	39.7 ± 2.6	----	44.2 ± 1.52	-----
	
	A	38.7 ± 2.7	0.750	41.5 ± 1.3	0.192	44.8 ± 0.91	0.715
	B	37.9 ± 3.1		40.4 ± 1.7		44.5 ± 1.24	
	C	38.8 ± 3.5		41.7 ± 2.1		45.6 ± 1.77	

**Length (Z-score)**	BM	-0.329 ± 0.903	----	-0.889 ± 0.972	----	-0.895 ± 0.845	----
	
	A	0.017 ± 1.260	0.386	-0.589 ± 0.272	0.784	-0.738 ± 0.354	0.700
	B	-0.619 ± 0.811		-0.807 ± 0.564		-0.537 ± 0.580	
	C	-0.215 ± 0.946		-0.642 ± 1.047		-0.441 ± 1.099	

**Head circumference** **(cm)**	BM	26.3 ± 1.3	----	28.0 ± 1.5	----	31.5 ± 1.28	-----
	
	A	27.7 ± 1.7	0.615	29.2 ± 1.1	0.418	32.1 ± 0.55	0.077
	B	26.8 ± 1.8		28.5 ± 1.3		32.3 ± 1.18	A-B: 0.033
	C	27.8 ± 3.2		28.7 ± 1.3		32.3 ± 0.75	A-C: 0.097
							B-C: 0.744

**Head circumference** **(Z-score)**	BM	-0.297 ± 0.820	----	-0.907 ± 0.831	----	-0.654 ± 0.553	---
	
	A	0.311 ± 1.375	0.164	-0.689 ± 0.429	0.409	-0.331 ± 0.517	0.203
	B	-0.511 ± 0.583		-0.858 ± 0.556		-0.472 ± 0.639	
	C	-0.433 ± 0.857		-1.066 ± 0.761		-0.671 ± 0.777	

**Fat mass (g)**	BM	----	----	111.1 ± 61.7	----	202.4 ± 49.6	----
	
	A	----	----	140.3 ± 72.5	0.131	193.4 ± 49.6	0.182
	B	----		135.7 ± 50.3		208.1 ± 61.0	
	C	----		129.9 ± 56.5		219.3 ± 52.0	

**Fat-free mass (g)**	BM	----	----	1190 ± 204	----	1699 ± 206	----
	
	A	----	----	1311 ± 158	0.925	1773 ± 152	0.007
	B	----		1168 ± 180		1790 ± 127	A-B: 0.009
	C	----		1274 ± 151		1915 ± 199	A-C: 0.044
							B-C: 0.277

**Body mass index** **(kg/m^2^)**	BM	8.16 ± 0.53	----	8.26 ± 0.69	----	16.0 ± 0.74	----
	
	A	7.61 ± 1.00	0.195	8.41 ± 1.06	0.424	16.8 ± 1.18	0.472
	B	8.37 ± 0.78		7.93 ± 0.80		15.6 ± 0.80	
	C	8.49 ± 1.25		8.04 ± 0.78		15.2 ± 1.57	

Number of patients in each group: 8 in A, 12 in B and 12 in C.

Data are presented as the means ± SD

* ONEWAY, with the factor group

** UNIANOVA, with the factor group and adjusted for covariates (initial corresponding value and duration of the intervention). If the p-value of the group < 0.1, pair comparisons have been performed.

At the end of the study, weight gain was greater in groups B and C than in group A (p = 0.002 and p = 0.002, respectively). In addition, groups B and C exhibited significant increases in weight z-scores compared to group A (p = 0.002 and p = 0.001, respectively). Length gains and head circumference gains were similar in all groups, but final head circumference was significantly higher in group B than in group A (p = 0.033). The greater weight gains observed in groups B and C were related to greater increases in FFM, which were significantly higher than in group A (p = 0.009 and p = 0.044, respectively).

Despite the greater protein intake in group C versus group B, there were no differences in weight gain or FFM accretion between these groups. Therefore, intake consisting of 150 kcal/kg/d of energy and 4.2 g/kg/d of protein, with a protein/energy ratio of 2.8 g/100 kcal, was sufficient to achieve appropriate increases in weight and FFM in VLBW infants during their hospital stays.

## Discussion

The breastfed group was regarded as a reference group for growth, FM accretion and FFM accretion. It was not considered in the statistical analysis because the intervention was only performed in formula-fed infants. Furthermore, the breastfed group happened to have the smallest children at birth. The weight differences at birth might have influenced the weight outcomes at the end of the study.

Our results show that an energy-enriched formula with a sufficient amount of protein increases weight gain with greater FFM accretion compared to normal formula in VLBW infants of appropriate weight for gestational age. The results were obtained after controlling for the initial values of weight gain and FFM accretion and for the duration of the intervention. Bioelectrical impedance is a straightforward, non-invasive, relatively inexpensive and portable method for evaluating changes in body composition [25]. Body composition was measured using BIA. This is not a common methodology and is subject to some inaccuracies because of the assumptions that need to be made in the equations that relate impedance to water content, from which FFM is estimated. However, it has been proven to be a valid method for assessing body composition in neonates [22,26].

The goal of nutrition in the VLBW infant is to optimize growth and neurodevelopmental outcomes while avoiding both short-term and long-term toxicity and adverse outcomes. Consistent with previous findings [14,27,28], our study noted greater weight gains in patients receiving high-energy intake than in those receiving standard-energy intake. Previously, we reported that administering a high-energy diet without increasing the amount of protein led to a disproportionate increase in body FM [14]. Our new results show that adding both protein and energy to an infant formula increases weight gain and improves weight Z-scores and leads to greater FFM accretion, without short-term clinical or analytical adverse effects. The increase in urea levels in this study was proportional to protein intake and was not clinically relevant. The weight gain and FFM accretion rates observed in groups B and C are similar to the changes described in fetuses between 32 and 35 weeks of gestation by Ziegler et al. [24]. Although the subjects in group C were fed more protein than those in group B (with protein/energy ratios of 3.1 g/100 kcal and 2.8 g/100 kcal, respectively), no improvement in terms of FFM accretion was observed. This observation was previously described by Fairey et al. [19] and could mean that protein intakes higher than 4.2 g/kg/d may exceed the capacity for protein utilization in VLBW infants, regardless of the accompanying energy intake; alternatively, higher energy intake may be required to improve protein utilization. Energy and protein intakes of 150 kcal/kg/day and 4.2 g/kg/day can be obtained with modular supplements added to a preterm formula, as we did in this study, or by increasing the volume or concentration of the product given to the infant.

In this early period of life, catch-up growth in head circumference was detected in each group, as indicated by the positive Z-score gains in all three groups. In addition, when the growth in head circumference was controlled for its initial value and for the duration of the intervention, it was statistically higher in group B than in group A. Postnatal head growth is an important clinical indicator of brain growth. In fact, poor postnatal head growth in preterm infants is strongly associated with poor neurodevelopmental outcomes and cerebral palsy [29]. Therefore, physicians caring for preterm infants should bear in mind that nutritional interventions aimed at limiting postnatal head growth restriction could improve neurodevelopmental outcomes [10,11,30-32].

Catch-up growth in intrauterine growth-restricted infants may increase their risk of obesity, hypertension, impaired glucose tolerance and cardiovascular disease. Therefore, there is a concern that accelerated growth during a critical period in preterm infants could lead to long-term adverse metabolic effects. A strength of our study is the inclusion of infants whose growth was appropriate for their gestational age, as these infants are metabolically different from intrauterine growth-restricted infants. The long-term effects of rapid growth of body weight on the onset of metabolic syndrome are relatively small compared to those of other risk factors (parental weight, lifestyle and growth later in childhood). These data suggest that the type and intake of nutrition needed by preterm infants with intrauterine growth restriction may be different from that of preterm infants with growth appropriate for their gestational age [33,34].

The macronutrient composition of breast milk was based on reports rather than directly measured; therefore, there may be some error in the estimated macronutrient intakes. Although we had the appropriate number of newborns in the high-protein and high-energy groups (based on the calculations for sample size), the number of newborns in the control group was less because four newborns were withdrawn from the study at the request of the parents. In addition, we were wary of administering high levels of protein to preterm infants due to the potential future risk of overweight or obesity, as has been reported in healthy, formula-fed infants [35]. In a group of subjects being fed infant formula with higher protein content, a larger increase in weight during the first two years of life was identified, with no effect on length [36].

## Conclusions

This study suggests that higher protein and energy intake during a critical period is advantageous for preterm infant growth and body composition because it increases weight gain, weight z-score and FFM accretion. Energy and protein intakes of 150 kcal/kg/d and 4.2 g/kg/d, respectively, are sufficient to increase FFM accretion.

## Competing interests

The authors declare that they have no competing interests.

## List of abbreviations

BIA: body electrical impedance analysis; BMI: body mass index; VLBW: very low birth weight; LBW: low birth weight; FFM: fat-free mass; FM: fat mass.

## Authors' contributions

JAC and JF were responsible for the study design and for writing the manuscript. JAC performed the anthropometrics measurements and BIA explorations. JF performed the statistical analysis. GR, RC and XC revised the manuscript. All the authors read and approved the final manuscript.
